# Oxeodaphne

**Published:** 1884-03

**Authors:** 


### OXEODAPHNE.

Dr. Edward Palmer, in his exhaustive article upon "The Plants used by Indians of the United States," says:

"*Oxeodaphne Californica.*—This fine evergreen tree of California has a very strong, spicy odor. By rubbing the hands and face a short time with the leaves a very distressing headache will be produced. Hahnemann is not the only discoverer of the fact that like cures like; for long before he was born, the Indians of California were aware of the power which this plant has of producing a headache in those that are well, and to cure those who are afflicted with it."—*Am. Nat.*, Vol. XII, p. 652.

J. Murray Moore has made enough of a proving of this drug to indicate that it would be very useful to us. (Allen, *Ency. Pure. Mat. Med.*, Vol. X, p. 609. "Oxeodaphne," *Month. Hom. Rev.*, Vol. XXII, p. 485.) Will not our California brothers give us a good, full proving? It should be done.

MILLSPAUGH.

