# Correction

**Published:** 1884-10

**Authors:** 


Correction.—Homœopathic Physician, Vol. IV, p. 69, *Oxeodaphne* should be *Oreodaphne*, ορεοδαφνε, Mountain Laurel. I pointed out the error to Dr. Murray Moore, a prover, and he acknowledged it, it having escaped his notice in proof-reading.

